# *Spartina alterniflora* invasion alters soil microbial community composition and microbial respiration following invasion chronosequence in a coastal wetland of China

**DOI:** 10.1038/srep26880

**Published:** 2016-05-31

**Authors:** Wen Yang, Nasreen Jeelani, Xin Leng, Xiaoli Cheng, Shuqing An

**Affiliations:** 1 School of Life Science and Institute of Wetland Ecology, Nanjing University, Nanjing 210093, P. R. China; 2 Key Laboratory of Aquatic Botany and Watershed Ecology, Wuhan Botanical Garden, Chinese Academy of Sciences, Wuhan 430074, P. R. China

## Abstract

The role of exotic plants in regulating soil microbial community structure and activity following invasion chronosequence remains unclear. We investigated soil microbial community structure and microbial respiration following *Spartina alterniflora* invasion in a chronosequence of 6-, 10-, 17-, and 20-year-old by comparing with bare flat in a coastal wetland of China. *S. alterniflora* invasion significantly increased soil moisture and salinity, the concentrations of soil water-soluble organic carbon and microbial biomass carbon (MBC), the quantities of total and various types of phospholipid fatty acids (PLFAs), the fungal:bacterial PLFAs ratio and cumulative microbial respiration compared with bare flat. The highest MBC, gram-negative bacterial and saturated straight-chain PLFAs were found in 10-year-old *S. alterniflora* soil, while the greatest total PLFAs, bacterial and gram-positive bacterial PLFAs were found in 10- and 17-year-old *S. alterniflora* soils. The monounsaturated:branched PLFAs ratio declined, and cumulative microbial respiration on a per-unit-PLFAs increased following *S. alterniflora* invasion in the chronosequence. Our results suggest that *S. alterniflora* invasion significantly increased the biomass of soil various microbial groups and microbial respiration compared to bare flat soil by increasing soil available substrate, and modifying soil physiochemical properties. Soil microbial community reached the most enriched condition in the 10-year-old *S. alterniflora* community.

Plant invasion, one component of anthropogenic-induced global change, has caused severe biological impacts on native ecosystems and great economic costs^1^ by changing the composition of species and the ecosystems’ structure^2^, processes and functioning^3^,^4^. Alterations in plant community structure may affect composition of soil microbial community and functioning by altering the quality and quantity of litter input and by modifying soil physical, chemical and biological environment^5^. Numerous studies have reported that plant invasion can alter the composition of the soil microbial community^6^,^7^,^8^, stimulate or inhibit microbial activity^9^,^10^, and change many important nutrient cycling processes and pools^4^,^11^. Nevertheless, our understanding of soil microbial community structure and activity as affected by plant invasion is still limited, particularly for different plant invasion chronosequences.

Plant invasion can influence soil microbial community structure and activity by altering the quantity and/or quality of litter entering the soil^11^,^12^. Previous studies have found that plant invasion can change aboveground (leaf litter) and belowground (root litter and exudates) inputs^13^,^14^. Elgersma *et al*.^8^ have reported that the alterations in the soil microbial community are mainly driven by belowground processes (e.g., belowground inputs) rather than aboveground litter inputs^8^. Plant invasion also shifts the resources available to soil microorganisms and further changes the soil microbial community^15^. Many invasive plants are considered to be more decay resistant owing to their higher levels of lignins, tannins, and other secondary compounds^12^, and lower quality of invasive plant materials (higher carbon (C)/nitrogen (N) ratio of litter and/or root)^14^. These invasive plants are ultimately the cause of higher soil C:N ratios relative to the native ecosystem^16^. The soil C:N ratio is a primary driver for the alteration in the soil microbial community structure^17^. The fungal biomass is highly associated with soil C:N ratio, whereas the bacterial biomass is negatively correlated with soil C:N ratio^17^,^18^. The response of the soil microbial community structure and activity to plant invasion exhibits high variation, probably as a result of the diverse changes of soil C sources and nutrient availability following invasion of distinct plant species^8^,^10^. Alteration in soil substrates during different periods (e.g., short-, mid-, and long-term) of plant invasion can also affect soil microbial community structure and activity^19^.

Plant invasion can modify the soil’s physical and chemical properties, such as soil moisture^16^,^20^, pH^21^, and salinity^16^,^20^, and further affect soil microbial community structure and activity^18^,^22^. Soil moisture is a decisive factor of C and N availability and plays a vital role in soil microbial community structure and activity^23^. The changes in soil moisture can cause alterations in the physiology and growth of some specific soil microbial groups^24^. Soil pH appears to be the main driving factor for the distribution of soil microorganisms, a higher soil pH would promote gram-negative (gram^−^) bacteria growth and reduce gram-positive (gram^+^) bacteria biomass^25^,^26^,^27^. Soil salinity has been considered as one of the important factors for affecting microbial community structure and activity, and high salinity can decrease soil osmotic potential, further affect microbial community composition, decrease microbial biomass and activity^28^. Thus, the changes in the soil substrates and physicochemical properties altogether affect the soil microbial community structure and activity following plant invasion in a chronosequence.

*Spartina alterniflora* is a perennial C_4_ grass plant that is native to North America. It has been introduced to China since 1979 for coastal erosion control and sediment stabilization^29^,^30^. *S. alterniflora* invasion in the coastal zone of China has expanded over the past 30 years, from Tianjin in the north to Beihai in the south, by occupying bare flat and/or by replacing native C_3_ plants (e.g., *Suaeda salsa* and *Phragmites australis*), and become one of the dominant plants in China’s coastal wetland^16^,^20^. Previous studies have reported that *S. alterniflora* has a longer growing season, a higher leaf area index and net photosynthetic rate, and a greater net primary production compared with the native plants, *Scirpus mariqueter* and *P. australis*^31^. Furthermore, *S. alterniflora* invasion significantly alters soil physicochemical properties^16^, soil organic C and N sequestration^14^,^29^, and emissions of greenhouse gases in the coastal wetland of eastern China^20^. However, little is known about the changes in the soil microbial community structure and activity in chronosequences following *S. alterniflora* invasion. We hypothesized that *S. alterniflora* invasion would alter soil microbial community structure and activity by changing soil C availability and physiochemical properties. To test this hypothesis, we determined soil phospholipid fatty acids (PLFAs) to analyze the soil microbial community structure, and determined cumulative microbial respiration, microbial respiration on a per-unit-PLFAs basis, and the respiration quotient (qCO_2_) after 30-days of incubation at 25 °C and 35 °C to analyze the soil microbial activity. We measured soil moisture, pH, salinity, soil organic C (SOC), soil organic N (SON), water-soluble organic carbon (WSOC), microbial biomass C (MBC), microbial biomass N (MBN), the MBC:MBN ratio, temperature sensitivity (Q_10_) of microbial respiration, and the aboveground and root biomass in invasive 6-, 10-, 17-, and 20-year-old *S. alterniflora* communities and compared these findings with those from a bare flat in a coastal wetland of China.

## Results

### Soil and plant properties

Soil moisture, salinity, WSOC, SOC, and SON in *S. alterniflora* soils were significantly higher than those in bare flat soil (Table 1). Soil moisture was the highest in 17- and 20-year-old *S. alterniflora* soils followed by 6- and 10-year-old *S. alterniflora* soils (Table 1). The pH in *S. alterniflora* soils were significantly lower than that in bare flat soil with the lowest pH in 6- and 17-year-old *S. alterniflora* soils (Table 1). The highest salinity and the lowest WSOC were found in 20-year-old *S. alterniflora* soil, while the greatest SOC concentration was found in 17-year-old *S. alterniflora* soil (Table 1). Aboveground biomass was the highest in the 17-year-old *S. alterniflora* community, followed by 20-, 10-, and 6-year-old *S. alterniflora* communities (Table 1). However, SON concentration and root biomass did not significantly change across the *S. alterniflora* invasion chronosequence (Table 1).

### Soil microbial biomass and structural diversity

**Figure 1:**
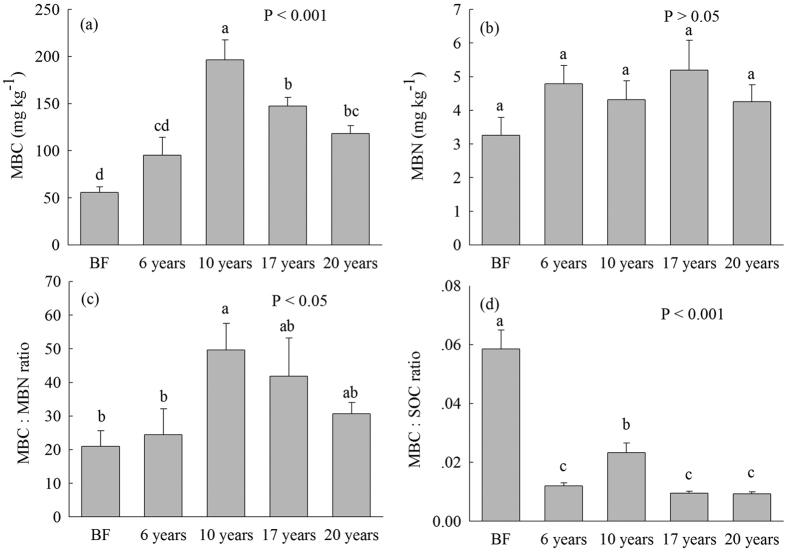
(**a**) Soil microbial biomass carbon (MBC), (**b**) Soil microbial biomass nitrogen (MBN), (**c**) MBC:MBN ratio and (**d**) MBC:SOC ratio (mean ± SE, n = 9) in bare flat (BF) and different invasion times (6, 10, 17 and 20 years) of *S. alterniflora* soils (0–30 cm depth). Different lower case letters over the bars indicate statistically significant differences at α = 0.05 level across the *S. alterniflora* invasion chronosequence.

The highest MBC concentration was found in 10-year-old *S. alterniflora* soil, followed by 17-, 20-, 6-year-old *S. alterniflora* and bare flat soils (Fig. 1a). In contrast, MBN did not significantly vary in the *S. alterniflora* invasion chronosequence (Fig. 1b). The MBC:MBN ratio in 10-year-old *S. alterniflora* soil was significantly higher than that in 6-year-old *S. alterniflora* and bare flat soils (Fig. 1c). The MBC:SOC ratio in bare flat soil was significantly higher than that in *S. alterniflora* soils, and the MBC:SOC ratio in 10-year-old *S. alterniflora* soil was significantly higher than that in 6-, 17-, 20-year-old *S. alterniflora* soils (Fig. 1d). MBC concentration was strongly associated with soil and plant properties except soil pH (Table 2).

**Figure 2:**
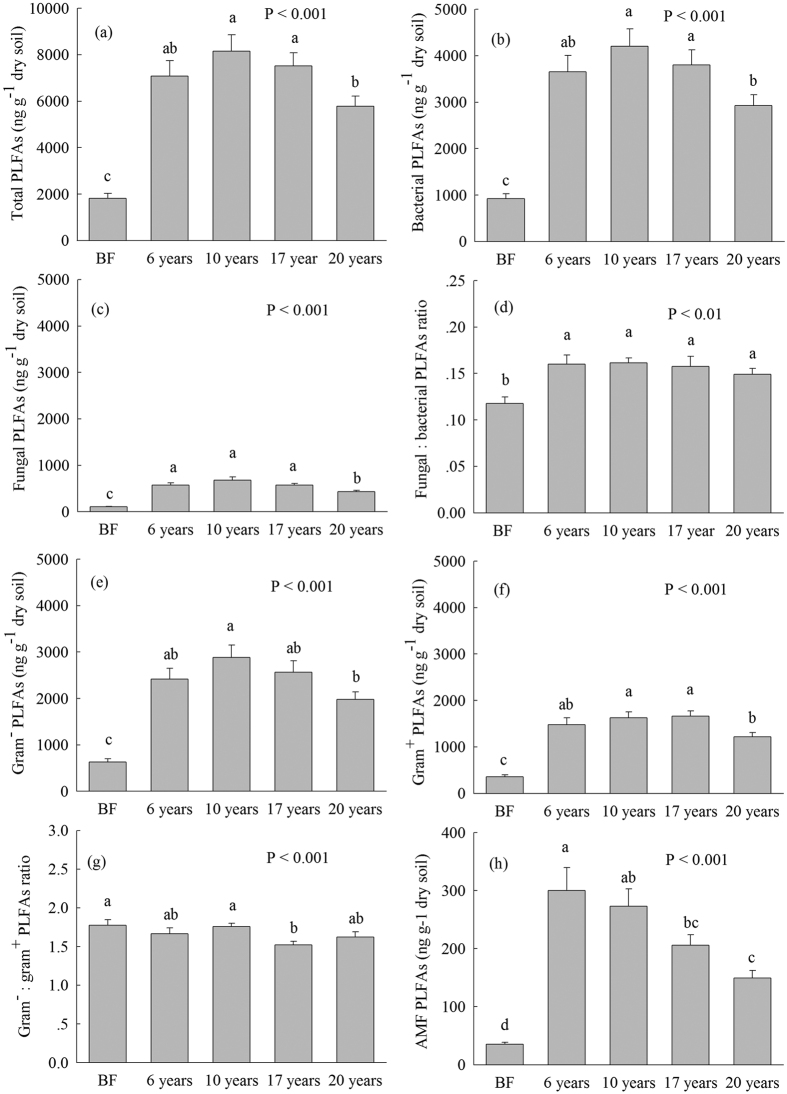
(**a**) The total phospholipid fatty acids (PLFAs), (**b**) Bacterial PLFAs, (**c**) Fungal PLFAs concentrations; (**d**) Fungal:bacterial PLFAs ratio; (**e**) Gram^−^ PLFAs, (**f**) Gram^+^ PLFAs concentrations, (**g**) Gram^−^:gram^+^ PLFAs ratio and (**h**) The arbuscular mycorrhizal fungal PLFAs (AMF PLFAs) concentration (mean ± SE, n = 9) in bare flat (BF) and different invasion times (6, 10, 17 and 20 years) of *S. alterniflora* soils (0–30 cm depth). Different lower case letters over the bars indicate statistically significant differences at α = 0.05 level across the *S. alterniflora* invasion chronosequence.

**Figure 3:**
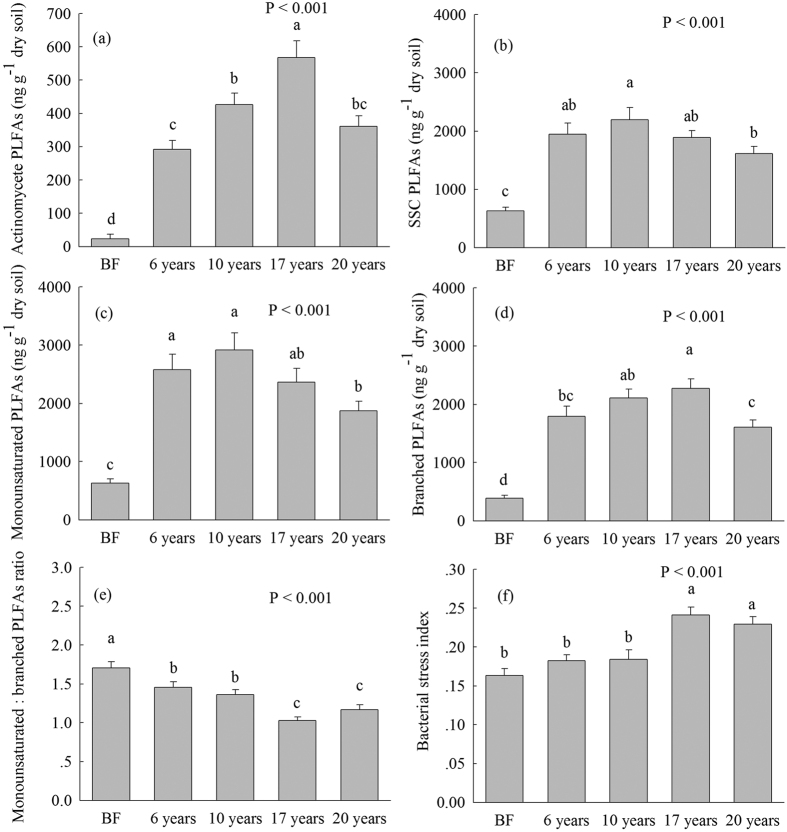
(**a**) Actinomycete phospholipid fatty acids (PLFAs), (**b**) The saturated straight-chain (SSC) PLFAs, (**c**) The monounsaturated PLFAs, (**d**) Branched PLFAs concentrations, (**e**) Monounsaturated:branched PLFAs ratio and (**f**) Bacterial stress index (mean ± SE, n = 9) in bare flat (BF) and different invasion times (6, 10, 17 and 20 years) of *S. alterniflora* soils (0–30 cm depth). Different lower case letters over the bars indicate statistically significant differences at α = 0.05 level across the *S. alterniflora* invasion chronosequence.

The quantities of the total PLFAs, bacterial, fungal, gram^+^ bacterial, gram^−^ bacterial, arbuscular mycorrhizal fungi (AMF), actinomycete, monounsaturated, branched, and saturated straight-chain (SSC) PLFAs in *S. alterniflora* soils were significantly higher than those in bare flat soil (Figs 2 and 3). The quantities of soil total PLFAs, bacterial, and gram^+^ bacterial PLFAs were the highest at 10 and 17 years, followed by 6 years and 20 years after *S. alterniflora* invasion (Fig. 2a,b,f). The lowest fungal PLFAs were found in 20-year-old *S. alterniflora* soil (Fig. 2c). The quantity of AMF PLFAs gradually declined following *S. alterniflora* invasion in the chronosequence (Fig. 2h). The quantities of actinomycete and branched PLFAs gradually increased from 6 to 17 years after *S. alterniflora* invasion, but declined in the 20-year-old *S. alterniflora* soil (Fig. 3a,d). The quantities of gram^−^ bacterial and SSC PLFAs were the greatest in 10-year-old *S. alterniflora* soil and the lowest in 20-year-old *S. alterniflora* soil (Figs 2e and 3b). The quantity of monounsaturated PLFAs was the most enriched in 10- and 6-year *S. alterniflora* soils, and it declined in 17- and 20-year *S. alterniflora* soils (Fig. 3c).

The fungal:bacterial PLFAs ratio in all *S. alterniflora* soils were considerably higher than that in bare flat soil (Fig. 2d). The lowest gram^−^:gram^+^ ratio was found in 17-year-old *S. alterniflora* soil, and the highest gram^−^:gram^+^ ratio was found in 10-year-old *S. alterniflora* and bare flat soils (Fig. 2g). The monounsaturated:branched PLFAs ratio in 17- and 20-year-old *S. alterniflora* soils were considerably lower than that in 6- and 10-year-old *S. alterniflora* soils (Fig. 3e). The bacterial stress index in 17- and 20-year-old *S. alterniflora* soils were significantly higher than that in 6- and 10-year-old *S. alterniflora* and bare flat soils (Fig. 3f).

### Soil microbial respiration and temperature sensitivity

**Figure 4:**
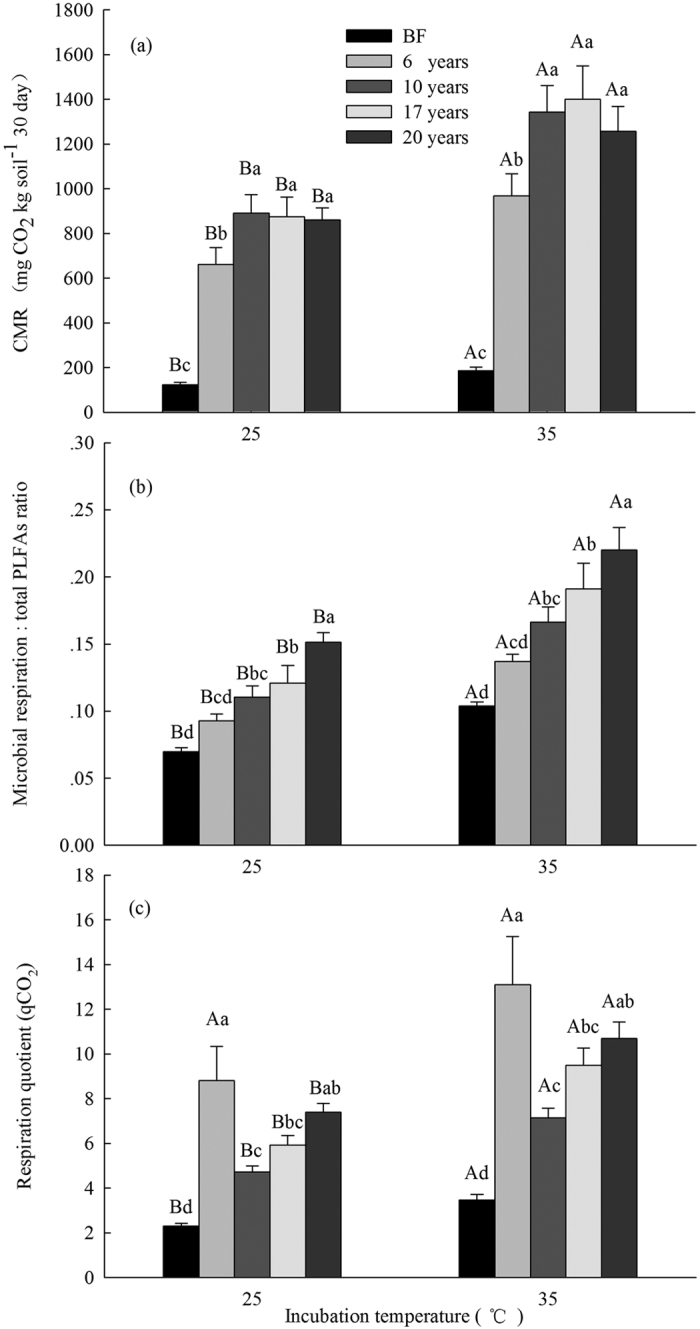
(**a**) Cumulative microbial respiration (CMR), (**b**) Microbial respiration on a per-unit-PLFAs basis and (**c**) Respiration quotient (qCO_2_) after 30-days incubation under different temperature treatments (25 °C and 35 °C) (mean ± SE, n = 9) in bare flat (BF) and different invasion times (6, 10, 17 and 20 years) of *S. alterniflora* soils (0–30 cm depth). Different lower case letters over the bars indicate statistically significant differences at α = 0.05 level across the *S. alterniflora* invasion chronosequence. Different upper case letters over the bars indicate statistically significant differences at α = 0.05 level between incubation temperature.

Cumulative microbial respiration in the 0–30 cm soil layer after 30 days of incubation at 35 °C was significantly greater than soil from the bare flat and *S. alterniflora* invasion chronosequence incubated at 25 °C (Fig. 4a). Cumulative microbial respiration in over 10-year-old *S. alterniflora* soils were considerably higher than that in 6-year-old *S. alterniflora* soil, which was higher compared with bare flat soil (Fig. 4a). Cumulative microbial respiration was not only significantly related to soil moisture, salinity, SOC, WSOC, SON, and aboveground and root biomass (Table 2) but also strongly associated with total and all types of PLFAs (Table 3).

**Figure 5:**
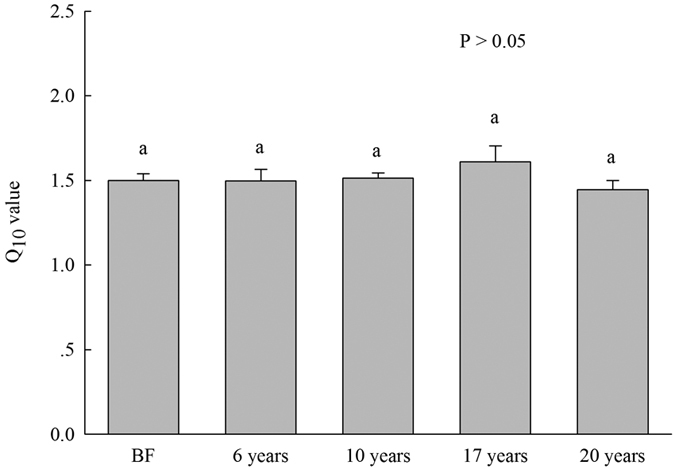
Temperature sensitivity (Q_10_) of microbial respiration after 30-days incubation time at 25 °C and 35 °C (mean ± SE, n = 9) in bare flat (BF) and different invasion times (6, 10, 17 and 20 years) of *S. alterniflora* soils (0–30 cm depth). Different lower case letters over the bars indicate statistically significant differences at α = 0.05 level across the *S. alterniflora* invasion chronosequence.

Similarly, microbial respiration on a per-unit-PLFAs and qCO_2_ after 30-days of incubation at 35 °C were significantly higher than in soils from all communities that were incubated at 25 °C, the exception being qCO_2_ in 6-year-old *S. alterniflora* soil (Fig. 4b,c). Microbial respiration on a per-unit-PLFAs gradually increased following the *S. alterniflora* invasion chronosequence (Fig. 4b). The qCO_2_ in 6- and 20-year-old *S. alterniflora* soils were significantly higher than that in 10-year-old *S. alterniflora* and bare flat soils (Fig. 4c). The Q_10_ value of microbial respiration did not significantly change across bare flat soil and the *S. alterniflora* invasion chronosequence (Fig. 5). Pearson’s correlation analysis showed that qCO_2_ was significantly associated with total and all types of PLFAs (Table 3) and that the Q_10_ value of microbial respiration was not significantly related to soil and plant properties (Table 2), and it was also unrelated to total and all types of PLFAs (Table 3).

### Controls on soil microbial community

**Figure 6:**
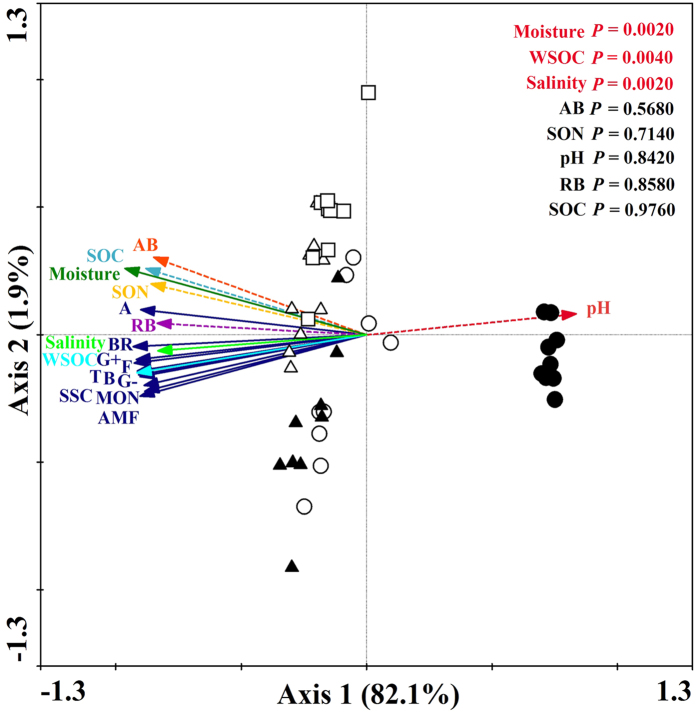
RDA results of PLFAs in the soil samples and soil and plant properties. The explanatory variables are showed by different arrows: PLFAs profiles by blue solid arrows: total PLFAs (T); bacterial PLFAs (B); fungal PLFAs (F); gram-positive bacterial PLFAs (G^+^); gram-negative bacterial PLFAs (G^−^); arbuscular mycorrhizal fungal PLFAs (AMF); actinomycete PLFAs (A); saturated straight-chain PLFAs (SSC), monounsaturated PLFAs (MON); branched PLFAs (BR); and the variables of soil and plant properties by colored arrow: soil moisture, pH, salinity, soil organic carbon (SOC), soil water-soluble organic carbon (WSOC), soil organic nitrogen (SON), aboveground biomass (AB) and root biomass (RB). Filled circles represent bare flat soil, open circles represent 6-year-old *S. alterniflora* soil, filled triangles represent 10-year-old *S. alterniflora* soil, open triangles represent 17-year-old *S. alterniflora* soil, and open squares represent 20-year-old *S. alterniflora* soil.

Eight variables of soil and plant properties, including, soil moisture, pH, salinity, SOC, WSOC, SON, aboveground and root biomass, explained 84.1% of the total variability in the PLFAs (Fig. 6). The variations in the PLFAs were strongly correlated with soil moisture (*F* = 131.16, *P* = 0.0020), WSOC (*F* = 8.75, *P* = 0.0040), and salinity (*F* = 10.07, *P* = 0.0020) (Fig. 6). The biggest variation, at 82.1%, was explained by the total variations of the PLFAs in Axis 1, and Axis 2 explained 1.9% of the total variations of the PLFAs (Fig. 6). Meanwhile, Pearson’s correlation analysis showed that the PLFAs were significantly positively correlated with soil moisture, salinity, SOC, WSOC, SON, aboveground and root biomass, but they were negatively associated with soil pH (Table 2).

## Discussion

Our findings not only added to various evidence that *S. alterniflora* invasion greatly accelerated soil organic C and N accumulation due to greater biomass input^14^,^29^ (Table 1), but also found that *S. alterniflora* invasion significantly increased MBC concentration and the quantities of the total and all types of PLFAs compared with bare flat soil (Figs 1, 2, 3). Soil C sources are considered as crucial ecological driving factors for microbial community dynamics^32^. Increased biomass input and the soil substrate following *S. alterniflora* invasion^14^,^16^ (Table 1) possibly enhanced MBC and all types of PLFAs. This speculation was supported by our Pearson’s correlation analysis that MBC and all types of PLFAs were highly associated with SOC, WSOC, SON, and above- and below- ground biomass (Table 2). The aboveground biomass and SOC content progressively increased in 6- to 17-year-old *S. alterniflora* soils and then fell in soils collected afterwards (Table 1). Interestingly, the highest MBC, gram^−^ bacterial and SSC PLFAs were found in 10-year-old *S. alterniflora* soil (Figs 1, 2, 3), and the greatest total PLFAs, bacterial and gram^+^ bacterial PLFAs were found in 10- and 17-year-old *S. alterniflora* soils (Fig. 2), implying that the soil microbial community reached the most enriched condition in 10-year-old *S. alterniflora* soil. The WSOC is the most important available substrate and directly provides available C and energy for soil microbial metabolism^33^,^34^. Although 17-year-old *S. alterniflora* soil had a bigger SOC stock compared with 10-year-old *S. alterniflora* soil (Table 1), there was no significant difference in the quantity of total PLFAs between 10- and 17-year-old *S. alterniflora* soils (Fig. 2) due to the same level of WSOC in both samples (Table 1). Meanwhile, the decrease in total PLFAs, bacterial, fungal, gram^+^ bacterial, and branched PLFAs in 20-year-old *S. alterniflora* soil compared with 10- and 17-year-old *S. alterniflora* soils (Figs 2 and 3), may be caused by lower levels of readily available substrate (i.e., WSOC; Table 1), which restricted soil microbial growth and metabolism. The RDA and Pearson’s correlation analysis confirmed that the variations in the all types of PLFAs were highly related to WSOC (Table 2, Fig. 6).

The fungal:bacterial PLFAs ratio can be used to reflect the physiological state of the soil microbial community that is particularly involved in SOM accumulation and turnover^35^, and the ecosystem’s buffering capacity^36^. In this study, *S. alterniflora* invasion significantly increased the fungal:bacterial PLFAs ratio compared with bare flat soil (Fig. 2d), indicating a higher C accumulation and self-buffering capacity in *S. alterniflora* soils. Previous studies reported that fungi have higher C assimilation efficiency relative to bacteria^37^,^38^, owing to their stronger ability to decompose plant compounds^39^,^40^. Higher C assimilation efficiency in fungi may result in more organic C being converted into more recalcitrant humic materials^37^. Hence, the increased fungal:bacterial PLFAs ratio in *S. alterniflora* soil can possibly enhance soil organic C sequestration following *S. alterniflora* invasion.

We found that the gram^−^:gram^+^ PLFAs ratio ranged from 1.52 to 1.78 across *S. alterniflora* invasion chronosequence (Fig. 2g), suggesting that gram^−^ bacteria dominated in bare flat and *S. alterniflora* salt marsh and that there were copiotrophic condition in this coastal wetland ecosystem^22^. Previous studies showed that higher soil pH would increase gram^−^ bacteria and decrease gram^+^ bacteria^25^,^26^,^27^. 10-year-old *S. alterniflora* and bare flat soils had a higher pH compared with 17-year-old *S. alterniflora* soil (Table 1), and this may be one of the reasons that the lowest gram^−^:gram^+^ PLFAs ratio was found in 17-year-old *S. alterniflora* soil (Fig. 2g). This result was consistent with our finding that 17- and 20-year-old *S. alterniflora* soils had a greater bacterial stress index than 10- and 6-year-old *S. alterniflora* and bare flat soils (Fig. 3f). Generally, a high bacterial stress index represents a slow rate of growth and long turnover time for gram^−^ bacteria^41^. Thus, higher bacterial stress index in 17- and 20-year-old *S. alterniflora* soils indicated that they had slower growth rates and lower turnover rates of the gram^−^ bacteria community relative to 10- and 6-year-old *S. alterniflora* and bare flat soils (Fig. 3f). Additionally, previous studies have reported that gram^−^ bacteria preferentially utilize fresh plant residual as an available C source, while gram^+^ bacteria prefer to use older, humified and more microbially processed SOM^42^,^43^. Thus, the lowest gram^−^:gram^+^ PLFAs ratio and the highest bacterial stress index were found in 17-year-old *S. alterniflora* soil (Figs 2g and 3f), indirect suggesting increased degree of SOM decomposition and humification in 17-year-old *S. alterniflora* soil compared with 10-year-old *S. alterniflora* and bare flat soils.

The monounsaturated and branched PLFAs were generally used to indicate aerobic and anaerobic microorganism biomass, respectively^41^,^44^. We found that the highest levels of monounsaturated PLFAs were found in 10- and 6-year-old *S. alterniflora* soils (Fig. 3c), while the highest levels of branched PLFAs was found in 17-year-old *S. alterniflora* soil across the invasion chronosequence (Fig. 3d), suggesting that the quantity of aerobic microbes was the highest in the early stage of *S. alterniflora* invasion, whereas the quantity of anaerobic microbes reached maximum levels at the later stage of *S. alterniflora* invasion. The ratio of monounsaturated:branched PLFAs continually declined during the invasion chronosequence (Fig. 3e), implying that the percent of anaerobic microbes gradually increased and the percent of aerobic microbes gradually decreased during the invasion chronosequence. This may be highly associated with the gradual increase in soil moisture during the invasion chronosequence (Table 1). Higher soil moisture provides stronger soil anaerobic conditions, which might be more suitable for anaerobic microorganism growth and facilitate SOM accumulation^45^,^46^.

Soil microbial respiration was highly dependent on soil temperature, moisture, and C inputs^47^, and was strongly associated with the quantities of the soil microbes and WSOC concentration^34^. In this study, cumulative microbial respiration at 25 °C and 35 °C at different invasion times of *S. alterniflora* soils were significantly higher than that in the bare flat (Fig. 4a), which was highly correlated with total and various types of PLFAs, WSOC, aboveground and root biomass (Tables 2 and 3). Thus, the increased cumulative microbial respiration in *S. alterniflora* soils may be greatly attributed to higher C inputs, and increase in available substrate (e.g., WSOC) and the microbial biomass (Table 1; Figs 1, 2, 3, 4). We found that cumulative microbial respiration and the microbial respiration:total PLFAs ratio at 35 °C in all communities were significantly higher than that at 25 °C (Fig. 4a,b), primarily because elevated temperature increases soil enzyme activities and further drives SOM decomposition^48^. Interestingly, the Q_10_ value of microbial respiration showed no obvious changes during the *S. alterniflora* invasion chronosequence (Fig. 5), likely because the Q_10_ of microbial respiration is not influenced by the differences in the microflora^34^ (Table 3). Although cumulative microbial respiration at 25 °C and 35 °C showed no significant differences between 10-, 17-, and 20-year-old *S. alterniflora* soils (Fig. 4a), cumulative microbial respiration on a per-unit-PLFAs basis at 25 °C and 35 °C progressively increased following the increase of invasion time (Fig. 4b), suggesting that *S. alterniflora* invasion may decrease microbial C utilization efficiency and enhance respiration loss in this coastal wetland ecosystem^48^. Generally, an increase in qCO_2_ may reflect a decrease in microbial C utilization efficiency and ecosystem stabilization^36^,^49^, and a higher MBC:SOC ratio could indicate an increase in microbes use C efficiency^16^,^50^. In this study, the lowest qCO_2_ and the highest MBC:SOC ratio were found in 10-year-old *S. alterniflora* soil following invasion from 6 to 20 years (Figs 1d and 4c), indicating that 10-year-old *S. alterniflora* soil had the highest microbial C utilization efficiency and the greatest ecosystem stabilization following invasion from 6 to 20 years. In addition, bare flat soil had higher the MBC:SOC ratio and lower cumulative microbial respiration on a per-unit-PLFAs and qCO_2_ relative to *S. alterniflora* soils (Figs 1d and 4b,c), implying that *S. alterniflora* invasion resulted in low microbial C utilization efficiency compared to bare flat^16^,^50^, which may be due to *S. alterniflora* with lower quality and more recalcitrant substance (e.g., lignin) is difficult to be utilized by microbes^14^.

In conclusion, this study highlighted the variations of soil microbial community structure and activity following bare flat was converted to *S. alterniflora* salt marsh in a invasion chronosequence in a coastal wetland of China. Specifically, *S. alterniflora* invasion greatly increased the total and various types of soil microbial biomass and cumulative microbial respiration but significantly decreased microbial C utilization efficiency compared to bare flat. 10-year-old *S. alterniflora* community had the most enriched soil microbial community and the highest microbial C utilization efficiency across 6 to 20 years *S. alterniflora* invasion. Soil microbial biomass decreased after 17 years *S. alterniflora* invasion. The variations in the microbial community structure and activity may in turn deeply affect SOM accumulation and ecosystem C and N cycling. This study represents a step forward in our understanding of microbial communities as affected by plant invasion, and it provides valuable insights regarding the better understand the influence mechanism of plant invasion on soil organic C pool.

## Methods

### Site description and sampling

This study was conducted at the core area of the Jiangsu Yancheng Wetland National Nature Reserve, Rare Birds, China (JYWNNRRB) (32°48′47″–34°29′28″N, and 119°53′45″–121°18′12″E). This area is characterized as warm temperate with an average annual temperature of 13.8 °C, average annual precipitation of 1000 mm, and average annual sea water salinity of 3.09%^16^. JYWNNRRB was designated as an internationally important wetland site (Ramsar) in 2002. *S. alterniflora* was introduced to the bare flat of the JYWNNRRB in 1983, and it quickly expanded to form large areas of *S. alterniflora* salt marshes following mudflat aggrading^14^. The bare flat and *S. alterniflora* salt marshes are located on the low and middle areas of the intertidal zone with semidiurnal tidal periodicity^20^. The seaward invasion region of *S. alterniflora* is a bare flat that had no vegetation prior to *S. alterniflora* invasion^16^.

The sampling region, with its different *S. alterniflora* invasion times, was identified based on analyses of Landsat Thematic Mapper 5 (TM5) satellite images and historical records. This chronosequence from seaward to landward contained the bare flat (BF) and the four *S. alterniflora* communities that were colonized in 2006 (6 years), 2002 (10 years), 1995 (17 years) and 1992 (20 years). In November 2012, three parallel transects (2-km length and 200-m width) were selected along the chronosequence. Within each transect, five locations were marked from the bare flat to the invasive 6, 10, 17, and 20 years *S. alterniflora* communities. Three 2 m × 2 m plots were randomly established within each location. Three soil samples (5-cm diameter × 30 cm depth) were collected randomly in each plot. The soil samples from each plot were mixed evenly to form a composite sample. Three 50 cm × 50 cm quadrats were established to collect aboveground biomass (i.e., the sum of leaves, stems and litter), and three root sampling blocks (15-cm length × 15-cm width × 30 cm depth) were excavated to collect root biomass in each community of each transect. All soil and plant samples were stored at 4 °C in the field and then transported to the laboratory for subsequent analysis.

### Laboratory analysis

Each root sampling block was put through a 100 mesh sieve and flushed with water, and the roots remaining in the sieve were collected at the final step^14^. All plant samples were carefully cleaned and oven-dried at 65 °C for determining aboveground and root biomass. The visible plant and fauna residues were removed from the soil samples, and soil samples were then divided into three subsamples after thorough mixing. One subsample was air-dried and passed through 1-mm sieves to measure soil pH, salinity, SOC and SON. A subsample of 2-mm sieved fresh soil was stored at 4 °C to determine WSOC, MBC, MBN and microbial respiration. Another subsample was passed through 2-mm sieves and stored at −80 °C as quickly as possible after freeze-drying and was used to analyze for PLFAs. The soil subsample was weighed and oven-dried at 105 °C to determine soil moisture. Soil pH was measured in a soil–water suspension (1:2.5 soil:water) with a glass electrode. Soil salinity was determined in a soil–water suspension (1:5 soil:water) with a conductivity meter. Before the SOC and SON analyses, approximately 10 g of dried soil subsamples were treated with 1 M HCl at room temperature for 24 h to eliminate total inorganic C and N^29^, and unhydrolyzed residues were analyzed with a CN elemental analyzer (Vario PYRO cube elemental analyzer, Germany) to obtain SOC and SON concentrations^14^. WSOC was determined using the method described by Yang *et al*.^16^. Briefly, WSOC was extracted from 10 g moist soil samples after addition of 20 mL distilled water. The extracted fluid was vacuum filtered through a 0.45 μm filter, and C concentration of the filtrate was rapidly determined by a Liqui TOCII analyzer (Elementar Analysensystem GmbH, Germany).

MBC and MBN were measured using the chloroform fumigation-extraction method^51^. 25 g dry-weight-equivalent of moist soil was fumigated with ethanol-free chloroform for 48 h at 25 °C in the dark. The fumigated and un-fumigated samples were then extracted with 100 mL 0.5 M K_2_SO_4_ by shaking for 30 min at 200 rpm and then filtered. Organic C and TN in the K_2_SO_4_-extracted solution were determined using a Liqui TOCII analyzer and the Kjeldahl method, respectively. MBC and MBN were calculated according to the equation: MBC = Ec/0.38, MBN = En/0.54, where Ec and En were organic C and TN extracted from fumigated soil subtracted organic C and TN extracted from unfumigated soil, respectively.

### Phospholipid fatty acids analysis

The soil microbial community composition was assessed using PLFAs analysis based on the method of Bossio and Scow^52^. Briefly, lipids were extracted from 8 g of a dry-weight-equivalent of the fresh soil subsample using 23 mL of an extraction mixture of chloroform: methanol: phosphate buffer (1:2:0.8, v/v/v). Phospholipids were then split into neutral, glyco- and phospho- lipids using solid-phase extraction columns by eluting with CHCl_3_, acetone and methanol, respectively. Subsequently, phospholipids were subjected to a mild-alkali methanolysis to recover fatty acid methyl esters. Samples were then re-dissolved in 200 ml hexane containing nonadecanoic acid methyl ester (19:0) as an internal standard and were analyzed using a Hewlett-Packard 6890 Gas Chromatograph equipped with an Ultra 2-methylpolysiloxane column with N_2_ as the carrier gas and H_2_ and air to support the flame. A 2-μL injection of the above dilution with a 1:50 split was employed at 250 °C for the injector and 300 °C for the detector. The oven temperature ramped from 170 °C to 300 °C at 5 °C min^−1^ and was held for 12 min. Peaks were identified using bacterial fatty acid standards and MIDI peak identification software (MIDI, Newark, DE). The quantity (ng g^−1^ dry soil) of each PLFAs was calculated based on the 19:0 internal standard (5 μg mL^−1^). The quantities of the PLFAs in each sample were expressed as ng PLFAs g^−1^ dry soil and were used to estimate microbial biomass. Bacteria were represented by the sum of the PLFAs: i14:0, i15:0, a15:0, 15:0, i16:0, i17:0, a17:0, 17:0, cy17:0, 14:1ω5c, 15:1ω6c, 16:1ω7c, and 18:1ω7c^44^,^52^,^53^. Fungi were represented by the sum of the PLFAs: 18:1ω9c, 18:2ω6,9c, and 20:1ω9c^53^,^54^,^55^. Gram^+^ bacteria were identified by the PLFAs: i13:0, i14:0, i15:0, a15:0, i16:0, a16:0, i17:0, and a17:0, and gram^−^ bacteria were identified by the PLFAs: 14:1ω5c, 15:1ω6c, 16:1ω7, 16:1ω9c, 17:1ω8c, 18:1ω7c, 12:0 2OH, 15:0 3OH, 16:1 2OH, cy17:0, cy19:0 ω8c, and 18:1ω7c 11-methyl^44^,^54^,^56^. AMF were identified by the PLFAs 16:1ω5c^44^,^54^,^57^. Actinomycete were identified by the PLFAs 10me 16:0 and 10me 17:0^41^. Monounsaturated PLFAs were estimated from the sum of the following PLFAs: 14:1ω5c, 15:1ω6c, 16:1ω5c, 16:1ω7c, 16:1ω9c, 17:1ω8c, 18:1ω7c, 18:1ω9c, and 20:1ω9c^21^,^41^,^44^. Branched PLFAs were estimated from the sum of following PLFAs: i13:0, i14:0, i15:0, a15:0, i16:0, a16:0, i17:0, a17:0, 10me 16:0, 10me 17:0, 12:0 2OH, 15:0 3OH, and16:1 2OH^41^,^44^,^52^. SSC PLFAs were estimated from the sum of the PLFAs: 12:0, 13:0, 14:0, 15:0, 16:0, 17:0, 18:0, and 20:0^44^,^52^. The total PLFAs of the microbial community were represented by the sum of fungal PLFAs, gram^+^ bacterial PLFAs, gram^−^ bacterial PLFAs, AMF PLFAs, actinomycete PLFAs, SSC PLFAs, and 20:4ω6,9,12,15c. The ratios of fungal:bacterial PLFAs, gram^−^:gram^+^ and monounsaturated:branched PLFAs were calculated from the above PLFAs. The ratio of cy17:0/16:1ω7c was used as a bacterial stress index, which indicates the growth stage of the gram^−^ bacteria community^58^.

### Microbial respiration measurements

Microbial respiration was measured by alkali absorption of CO_2_ evolved at 25 °C and 35 °C for 30 days in a laboratory aerobic incubation experiment with soil^37^,^48^. Briefly, the fresh soil sample (20 g dry weight equivalent) was evenly placed in a 50 mL glass beaker. Distilled water was added to the soil samples to maintain moisture at 60% of water-holding capacity. The glass beaker was placed in a 500 mL mason jar, and the glass tubes containing 10 mL 0.5 M NaOH solution was placed in each mason jar to capture CO_2_ evolved by the soil in the mason jar. The mason jar was sealed and incubated at 25 °C and 35 °C in the dark for 30 days. After incubation for 6, 12, 18, 24, and 30 days, the glass tubes that were equipped with NaOH were removed, and the mason jar was opened for several minutes to maintain sufficient O_2_ levels. The amount of CO_2_ was determined by titration of the NaOH solution with 0.1 M HCl in two drops BaCl_2_.

Temperature sensitivity (Q_10_) of microbial respiration was determined using equation (1)^48^,^59^.


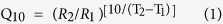


where R_2_ and R_1_ are the mean microbial respiration rate at T_2_ (35 °C) and T_1_ (25 °C), which are the temperature levels within 30 days of incubation.

The microbial respiration quotient (qCO_2_) was calculated by dividing the microbial respiration (mg CO_2_ 30 day) per kg by the MBC^36^.

### Statistical analyses

All of the statistical analyses were performed using SPSS Statistics 19 software. Data not meeting assumptions of normality and homogeneity of variance were log- or cube root-converted prior to statistical testing. One-way analysis of variance (ANOVA) was used to determine the statistical significance of the effect of *S. alterniflora* invasion time on soil and plant properties, microbial biomass and various types of PLFAs, cumulative microbial respiration, microbial respiration:total PLFAs ratio, qCO_2_ and Q_10_. One-way ANOVA was also used to determine the statistical significance of the incubation temperatures on cumulative microbial respiration, the ratio of microbial respiration:total PLFAs and qCO_2_. Pearson’s correlation analysis was performed to correlate soil microbial indexes with the soil and plant characteristics, and to correlate soil microbial respiration indexes with microbial biomass (i.e., each of the PLFAs). Soil and plant characteristics were tested for significant contributions to explain the variations in the PLFAs data with redundancy analysis (RDA) using CANOCO software for Windows 4.5. The statistical significance of the RDA was tested using the Monte Carlo permutation test (499 permutations; *P* < 0.05).

## Additional Information

**How to cite this article**: Yang, W. *et al*. *Spartina alterniflora* invasion alters soil microbial community composition and microbial respiration following invasion chronosequence in a coastal wetland of China. *Sci. Rep*. **6**, 26880; doi: 10.1038/srep26880 (2016).

## Figures and Tables

**Table 1:** Soil (0–30 cm depth) and plant properties (mean ± SE, n = 9) following *S. alterniflora* invasion in a coastal wetland of China.

	Moisture (%)	pH	Salinity (%)	SOC (g kg^−1^)	WSOC (mg kg^−1^)	SON (g kg^−1^)	Aboveground biomass (g m^−2^)	Root biomass (g m^−2^)
Bare flat	19.67 ± 0.37^c^	8.87 ± 0.02^a^	0.66 ± 0.04^c^	0.95 ± 0.02^c^	28.33 ± 0.34^d^	0.219 ± 0.035^b^	–	–
*S. alterniflora*								
6 years	45.45 ± 0.86^b^	8.48 ± 0.04^c^	1.82 ± 0.20^b^	10.07 ± 1.01^b^	55.62 ± 0.38^b^	1.019 ± 0.168^a^	1777 ± 137^c^	5530 ± 468^a^
10 years	46.57 ± 0.47^b^	8.59 ± 0.03^b^	1.85 ± 0.17^b^	10.25 ± 1.92^b^	61.92 ± 1.74^a^	1.058 ± 0.245^a^	1845 ± 138^c^	5808 ± 601^a^
17 years	52.54 ± 0.39^a^	8.46 ± 0.03^c^	1.78 ± 0.10^b^	15.56 ± 0.50^a^	62.14 ± 1.01^a^	1.357 ± 0.039^a^	3009 ± 175^a^	5291 ± 269^a^
20 years	51.17 ± 0.33^a^	8.54 ± 0.03^bc^	2.23 ± 0.07^a^	11.92 ± 0.64^ab^	47.74 ± 1.19^c^	1.135 ± 0.039^a^	2330 ± 116^b^	5435 ± 707^a^
Source of variation								
Invasion time	^***^	^***^	^***^	^***^	^**^	^**^	^***^	n.s.

Different letters indicate statistically significant differences at α = 0.05 level across the *S. alterniflora* invasion chronosequence. **P < 0.01; ***P < 0.001; n.s.: not significant; SOC: soil organic carbon; WSOC: soil water-soluble organic carbon; SON: soil organic nitrogen.

**Table 2:** Pearson correlation coefficients between soil microbial indexes and the soil and plant properties across the communities.

	Moisture	pH	Salinity	SOC	WSOC	SON	Aboveground biomass	Root biomass
MBC	0.563^**^	−0.469^**^	0.479^**^	0.483^**^	0.747^**^	0.480^**^	0.498^**^	0.569^**^
MBN	0.263	−0.343^*^	0.238	0.235	0.308^*^	0.199	0.326^*^	0.336^*^
MBC:MBN	0.281	−0.144	0.252	0.333^*^	0.381^**^	0.344^*^	0.192	0.215
Total PLFAs	0.754^**^	−0.720^**^	0.734^**^	0.676^**^	0.796^**^	0.674^**^	0.651^**^	0.649^**^
Bacterial PLFAs	0.752^**^	−0.713^**^	0.726^**^	0.658^**^	0.785^**^	0.660^**^	0.631^**^	0.642^**^
Fungal PLFAs	0.660^**^	−0.658^**^	0.683^**^	0.644^**^	0.801^**^	0.640^**^	0.621^**^	0.656^**^
Gram^+^ bacterial PLFAs	0.781^**^	−0.751^**^	0.735^**^	0.718^**^	0.812^**^	0.705^**^	0.698^**^	0.661^**^
Gram^−^ bacterial PLFAs	0.736^**^	−0.696^**^	0.714^**^	0.649^**^	0.774^**^	0.653^**^	0.621^**^	0.619^**^
AMF PLFAs	0.687^**^	−0.687^**^	0.678^**^	0.504^**^	0.689^**^	0.540^**^	0.461^**^	0.581^**^
Actinomycete PLFAs	0.680^**^	−0.664^**^	0.615^**^	0.798^**^	0.789^**^	0.747^**^	0.791^**^	0.614^**^
Monounsaturated PLFAs	0.711^**^	−0.679^**^	0.706^**^	0.583^**^	0.737^**^	0.600^**^	0.548^**^	0.600^**^
Branched PLFAs	0.772^**^	−0.743^**^	0.719^**^	0.757^**^	0.832^**^	0.733^**^	0.740^**^	0.673^**^
SSC PLFAs	0.752^**^	−0.700^**^	0.756^**^	0.618^**^	0.747^**^	0.626^**^	0.592^**^	0.634^**^
CMR at 25 °C	0.911^**^	−0.796^**^	0.876^**^	0.735^**^	0.718^**^	0.704^**^	0.738^**^	0.688^**^
CMR at 35 °C	0.901^**^	−0.775^**^	0.822^**^	0.750^**^	0.713^**^	0.721^**^	0.736^**^	0.656^**^
Q_10_ value	0.025	−0.025	−0.123	0.160	0.103	0.180	0.083	0.008

^*^P < 0.05; ^**^P < 0.01. MBC: microbial biomass carbon; MBN: microbial biomass nitrogen; PLFAs: phospholipid fatty acids; Gram^+^: gram-positive; Gram^−^: gram-negative; AMF: arbuscular mycorrhizal fungal; SSC: saturated straight-chain; CMR: cumulative microbial respiration; Q_10_: temperature sensitivity; See Table 1 for abbreviations.

**Table 3:** Pearson correlation coefficients between soil microbial respiration and microbial biomass across the communities.

	Total PLFAs	Bacterial PLFAs	Fungal PLFAs	Gram^+^ bacterial PLFAs	Gram^−^ bacterial PLFAs	AMF PLFAs	Actinomycete PLFAs	Monounsaturated PLFAs	Branched PLFAs	SSC PLFAs
CMR at 25 °C	0.811^**^	0.808^**^	0.748^**^	0.813^**^	0.800^**^	0.686^**^	0.729^**^	0.770^**^	0.813^**^	0.813^**^
CMR at 35 °C	0.817^**^	0.815^**^	0.735^**^	0.824^**^	0.812^**^	0.676^**^	0.772^**^	0.769^**^	0.832^**^	0.800^**^
MRP at 25 °C	0.210	0.201	0.184	0.236	0.191	0.091	0.291	0.148	0.258	0.217
MRP at 35 °C	0.249	0.241	0.200	0.283	0.234	0.112	0.370^*^	0.176	0.315^*^	0.234
qCO_2_ at 25 °C	0.486^**^	0.471^**^	0.419^**^	0.495^**^	0.465^**^	0.434^**^	0.453^**^	0.440^**^	0.491^**^	0.508^**^
qCO_2_ at 35 °C	0.510^**^	0.498^**^	0.425^**^	0.527^**^	0.494^**^	0.444^**^	0.511^**^	0.456^**^	0.531^**^	0.515^**^
Q_10_ value	0.128	0.133	0.056	0.151	0.146	0.060	0.253	0.097	0.180	0.058

^*^P < 0.05; ^**^P < 0.01. MRP: microbial respiration: total PLFAs ratio; qCO_2_: respiration quotient; See Table 2 for abbreviations.
